# Growth Conditions Determine the *DNF2* Requirement for Symbiosis

**DOI:** 10.1371/journal.pone.0091866

**Published:** 2014-03-14

**Authors:** Fathi Berrabah, Marie Bourcy, Anne Cayrel, Alexis Eschstruth, Samuel Mondy, Pascal Ratet, Benjamin Gourion

**Affiliations:** Institut des Sciences du Végétal, Centre National de la Recherche Scientifique, Gif sur Yvette, France; Ecole Normale Superieure, France

## Abstract

Rhizobia and legumes are able to interact in a symbiotic way leading to the development of root nodules. Within nodules, rhizobia fix nitrogen for the benefit of the plant. These interactions are efficient because spectacularly high densities of nitrogen fixing rhizobia are maintained in the plant cells. *DNF2*, a *Medicago truncatula* gene has been described as required for nitrogen fixation, bacteroid’s persistence and to prevent defense-like reactions in the nodules. This manuscript shows that a Rhizobium mutant unable to differentiate is not sufficient to trigger defense-like reactions in this organ. Furthermore, we show that the requirement of *DNF2* for effective symbiosis can be overcome by permissive growth conditions. The *dnf2* knockout mutants grown *in vitro* on agarose or Phytagel as gelling agents are able to produce nodules fixing nitrogen with the same efficiency as the wild-type. However, when agarose medium is supplemented with the plant defense elicitor ulvan, the *dnf2* mutant recovers the fix^−^ phenotype. Together, our data show that plant growth conditions impact the gene requirement for symbiotic nitrogen fixation and suggest that they influence the symbiotic suppression of defense reactions in nodules.

## Introduction

Legumes and rhizobia form symbiotic interactions leading to the development of a symbiotic organ (the nodule) in which bacteria fix atmospheric nitrogen for the benefit of the plant. Legume nodules are massively colonized by rhizobia and endosymbionts (bacteroids) accumulate into so called symbiotic cells. Despite this infection level, nodules do not display defense reactions suggesting that the innate immunity is suppressed in this organ in order to allow chronic infection by rhizobia [1]. This hypothesis is supported by the observation that when compared to the infected cells, uninfected nodule cells express a relatively high number of genes that can be associated with biotic stress or defense responses against pathogenic microbes [2]. Now that the transfer of symbiotic capacity to non-legume plants is considered as a feasible challenge [3], understanding how bacteroids are maintained at a high density in symbiotic cells during the rhizobium/legume symbiosis is crucial. Bacteroid maintenance defect is associated with two physiological processes; i) premature senescence of nodules that is frequently associated with nitrogen fixation defect and ii), defense-like reactions in the nodules. In the later situation, which is caused by either plant or bacterial mutants or inappropriate plant growth conditions for nodule development [4], it is unclear whether the defense-like reactions are the cause or the consequence of the symbiotic defect.

A key step in the comprehension of the plant tolerance to the massive and chronic rhizobia infection is the identification of plant genes involved in this process. Recently, a gene involved in bacteroid maintenance has been identified in *Medicago truncatula* (one of the favorite models used to study rhizobium/legume interactions) [5]. This gene, *DNF2*, encodes a phosphatidyl-inositol specific phospholipase C X domain containing protein [5]. The *dnf2* mutants are competent for the initial steps of the symbiosis but deficient for nitrogen fixation (nod^+^ fix^−^) [5]. In mutant nodules the bacteria are released into the plant cells where they rapidly loose viability [5]. Nodules of *dnf2* mutant display typical features of defense reactions such as phenolics accumulation [5], [6], and induction of a *PR10* gene, accompanied with necrosis [4], [5]. Furthermore, bacteroids terminal differentiation triggered in *M. truncatula* by antimicrobial nodule cysteine rich (NCR) plant peptides [7] is altered in the *dnf2* mutant [5]. It is not clear whether defense-like reactions in the *dnf2* mutant are a cause or a consequence of bacteroid differentiation defect.

The relationship between plant defenses and rhizobium/legume symbiosis has been previously investigated. However, all the studies were focused on the early steps of the symbiotic process [4], [8], [9], [10], [11], [12], [13], [14], [15] or done in cell-culture systems [10]. The potential effect of plant growth condition on the symbiotic control of endosymbiont persistence and of plant defenses after the internalization step of the symbiosis is not well understood [16]. In contrast, in the plant/pathogen interactions, the effect of (biotic) environment is well known to modulate plant defenses [10], [17], [18]. Here we present data indicating that plant growth conditions determine the *DNF2* requirement for chronic infection by rhizobia and nitrogen fixation and that no defense-like reactions are elicited in nodules of *dnf2* mutant growing under permissive conditions.

## Materials and Methods

### Plant and Bacterial Cultures

*Medicago truncatula* ecotypes R108 [19] and A17 [20] and the derived mutants [5], [21], [22], [23] were grown *in vitro* on Buffered Nodulation Medium (BNM) [24] supplemented with 1 µM AVG [L-a-(2-aminoethoxyvinyl)-Gly] and solidified with gelling agents as indicated. Agar HP 696–7470 was from Kalys (Bernin, France; http://www.kalys.com/), Bacto Agar from Difco (Sparks, USA), Agarose GEPAGA0765 from Eurobio (Courtaboeuf, France; http://www.eurobio.fr) and Phytagel from Sigma Aldrich (http://www.sigmaaldrich.com/). Agar and bactoagar were used at 2%, Phytagel and agarose at 0.8%. *Sinorhizobium meliloti* strain Rm41 [25], strain SM1021 [26] and *bacA* [27] and strain *Sinorhizobium medicae* strain WSM419 [28] were cultivated in YEB medium [29], *Pseudomonas fluorescens* Q2-87 [30] in King’s B medium [31] and *Bradyrhizobium* sp. ORS285 [32] on YM medium [33]. All these bacteria were cultivated at 30°C with shaking. *Escherichia coli* K12 was cultivated in LB medium at 37°C with shaking.

### Plant Inoculation

*M. truncatula* seeds were surface sterilized as previously described [5] and vernalized for at least 48 h at 4°C in the dark on water agarose (1%) plates. Seeds were then germinated by incubating them at 24°C for 36 h before being transferred to 12 cm square plates containing BNM. For single inoculation, overnight cultures of *S. medicae* were pelleted and washed in sterile water. OD_600 nm_ was then adjusted to 0.1 in water by re-suspension. Eight seedlings per plate were together inoculated with 1 mL of *S. medicae* cell suspensions. For co-inoculation experiments, *S. medicae* strain WSM419 and either one of the following strain: *E. coli* K12 or *Bradyrhizobium* sp. ORS278 or *P. fluorescens* Q2–87 were pelleted separately, washed in sterile water, re-suspended in water and the OD_600 nm_ were adjusted to 0.4 for WSM419 and 0.2 for other bacteria. Suspensions of *S. medicae* and one of the other strains were then mixed in a 1∶1 ratio (V/V) and the resulting suspension was used to inoculate seedlings as described above for single inoculation.

### Microscopy

For all microscopy imaging presented in this study, nodules were prepared as described in [5], [7]. They were either embedded into technovit matrix or in agarose (6%). From technovit embedded material thin sections (7 µm) were prepared using a microtome and stained with 0.02% toluidine blue. Agarose sections were made using a vibratome. For β-glucuronidase activity detection, roots were vacuum-infiltrated with X-gluc and treated as described [34]. Sections were observed and photographed with a Leica DMI 6000B inverted microscope. Phenolic compounds were revealed as described previously [35]. Agarose sections were observed using a macroscope Nikon AZ10.

### Acetylene Reduction Assays (ARA)

ARA were conducted on single plants using a protocol derived from [36]. Briefly, a single nodulated plant was placed into a 10 ml glass vial sealed with a rubber septum. 250 µl of acetylene were injected per vial. Plants were incubated for at least one hour at room temperature and gas samples (200 µl) were analyzed by gas chromatography using the 7820A Gas Chromatograph from Agilent Technologies (Santa Clara, USA) equipped with a flame ionization detector and a GS-Alumina column (50 m×0.53 mm) with hydrogen as carrier gas. Column temperature and gas flow rate were 120°C and 7.5 mL/min, respectively.

### Molecular Biology

RNAs were extracted using the RNeasy Plant Mini Kit (Qiagen, http://www.qiagen.com/). For cDNA synthesis, RNA samples were treated with RNase-free DNAse I to remove traces of genomic DNA. Reverse transcription (RT) was then performed from 1 µg of total RNA using a poly-T primer and the First Strand cDNA Synthesis Kit from Fermentas (http://www.fermentas.de) and RT-qPCR was performed as described in [5]. *MtACTIN2* was used as a reference gene. Primers are listed in Table S1.

### Statistical Analysis

All statistical analyses were performed on R2.14.2 (R-Development-Core-Team 2012).

## Results

### Lack of Bacteroid Differentiation is not Sufficient to Trigger Defense-like Reactions in Nodules

**Figure 1:**
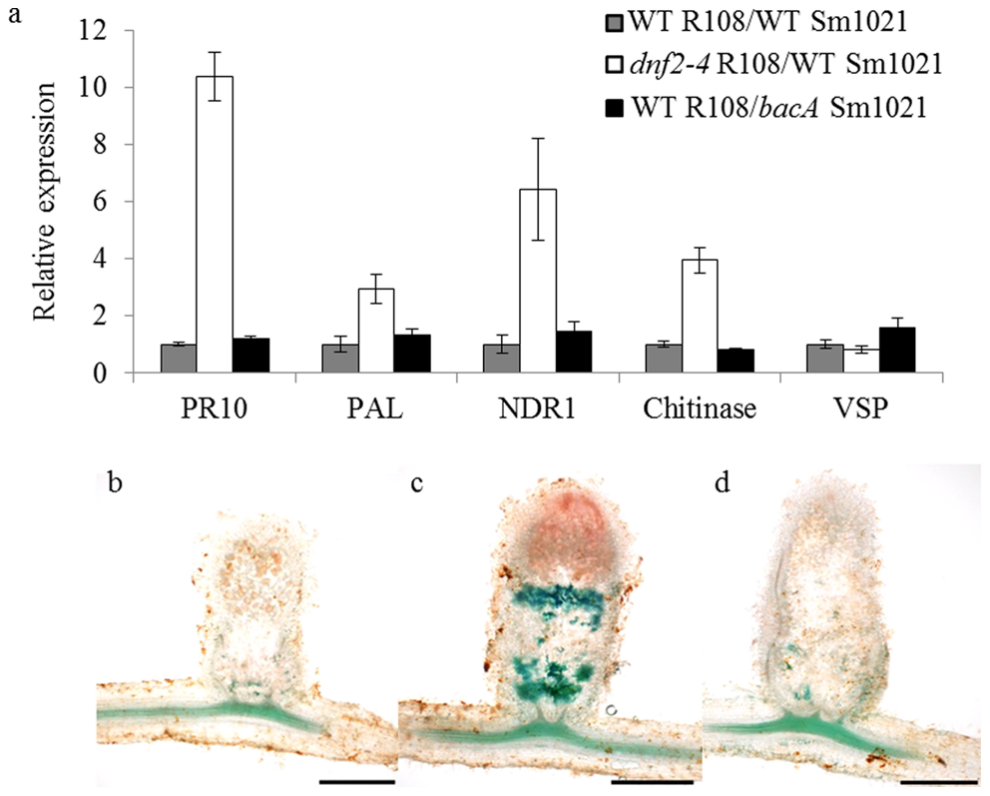
Bacteroid differentiation defect is not sufficient to trigger defense-like reactions in *dnf2* nodules. Panel a: Expression of defense markers was evaluated by RT-qPCR using cDNA prepared from 14 dpi nodules. The y axis represents fold induction/WT. Panel b: 25 dpi nodules of R108 WT induced by Sm1021 WT. Panel c: 25 dpi nodules of *dnf2–4* induced by Sm1021 WT. Panel d: 25 dpi nodules of R108 WT induced by a SM1021 *bacA* derivative. Nodules in b, c and d were stained for phenolics using potassium permanganate toluidine blue (scale bars 500 µm).

In order to better characterize the *dnf2* defense-like reactions phenotype, we examined the expression of defense related genes in *dnf2* nodules by RT-qPCR. In addition to *PR10* (TC94217/MTR_2g035150, [37]), expression of *phenylalanineamonialyase* (*PAL*, TC106667, [38]), *chitinase* (Medtr3g118390, [39]), *non-race specific disease resistance 1* (*NDR1*, Mtr.40876.1.S1_at, TC108235) [40], and *vesicle storage protein* (VSP, TC93960, [38]) was evaluated. Out of the five defense markers evaluated, four were found to be induced more than two fold in the *dnf2–4* nodules when compared to the wild-type (WT) nodules (Figure 1a). *dnf2* develops defense–like reactions and displays a bacteroid differentiation defect. In order to determine if differentiation defect triggers defense-like reactions, phenolics accumulation and defense-related gene expression were examined in nodules induced by the *S. meliloti bacA* mutant (Figure 1). *bacA* mutant is hypersensitive to NCRs [41] and rapidly degenerate inside symbiotic cells [42]. As a consequence, the *bacA* mutant does not undergo terminal differentiation and does not fix nitrogen. In contrast to what is observed in *dnf2–4* nodules (Figure 1c), WT nodules hosting *bacA* mutant did not accumulate phenolics (Figure 1d) similar to that of *M. truncatula* WT nodules induced by the WT bacteria (Figure 1b). Furthermore, defense related genes were not induced in the nodules induced by the *bacA* mutant (Figure 1a). These data indicate that lack of differentiation is not sufficient to elicit defense-like reactions in nodules.

### Plant Growth Conditions Impact the *dnf2* Phenotype

**Figure 2:**
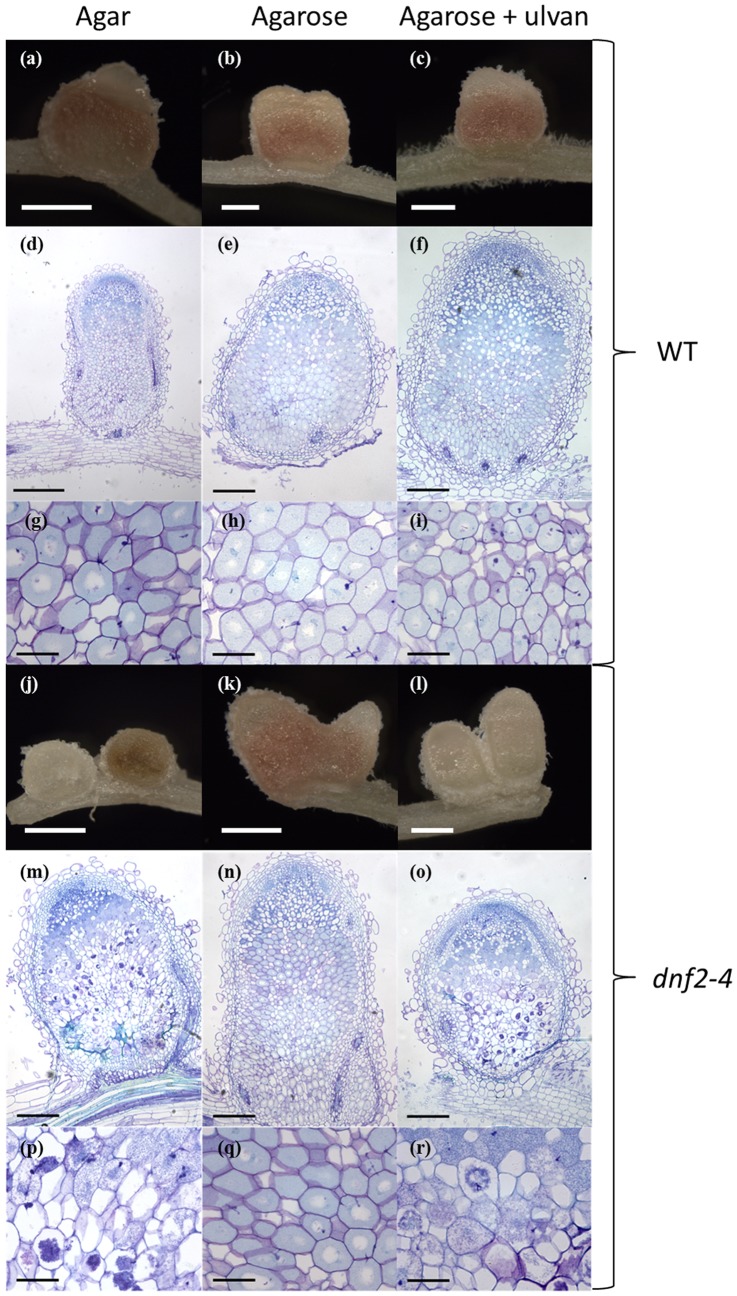
Plant growth conditions impact the *dnf2* phenotype. Nodules were harvested 18 days post inoculation with Rm41. a-i, Wild-type nodules. j-r, *dnf2–4* nodules. Left panels, middle panels and right panels represent nodules grown on agar-, agarose- and agarose-based BNM supplemented with 1% ulvan respectively. Panels a, b, c, j, k and l (scale bars 500 µm) illustrate whole nodules, panels d, e, f, m, n and o (scale bars 200 µm except for d 500 µm) are thin sections of whole nodules and panels g, h, i, p, q, r (scale bars 50 µm) are enlargement of the zone III of panels d, e, f, m, n and o, respectively.

We evaluated whether the plant growth condition had an impact on the *dnf2* mutant phenotype. For this we compared *dnf2–4* and wild-type nodules of plants developed on media solidified with either agar or agarose. Both agar and agarose are solidifying agents extracted from algae but agarose has a higher purity level, does not contain agaropectins and harbors a reduced level of undefined trace elements. In contrast to white and necrotic nodules previously observed on agar-conditions [5], when agarose was used to solidify the medium, *dnf2–4* plants produced a mixture of white and pink nodules (Figure 2k). The pink nodules were indistinguishable from nodules on WT plants (Figure 2b). Nodule occupancy and zonation were studied by imaging nodule sections of plants that were grown on different substrates. In contrast to the phenotype observed on plants cultivated on agar medium ([5]; Figure 2), on agarose-based medium nodule structures (Figure 2e,n) and infected cells (Figure 2h,q) were similar among WT and *dnf2–4* plants. These observations suggested that the *dnf2–4* mutant is a conditional mutant and the genetic requirement to prevent defense-like reactions, to accumulate leghemoglobin and to produce normally infected nodules can be modulated by the plant substrate.

### Plant Growth Conditions Determine the *DNF2* Requirement for Nitrogen Fixation

**Figure 3:**
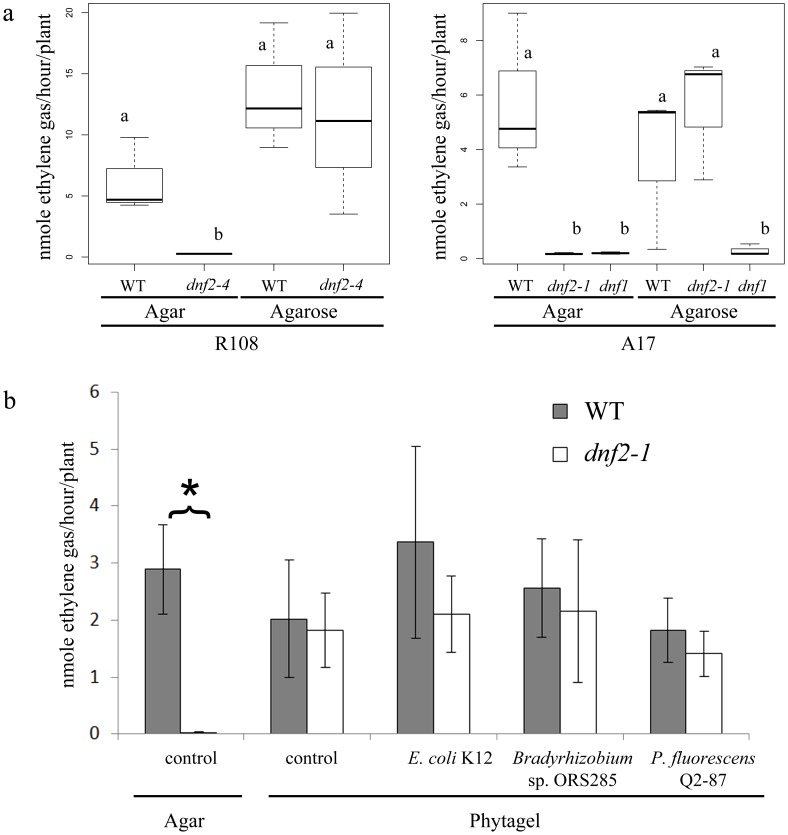
Plant growth conditions determine the *DNF2* requirement for symbiosis. a) Acetylene reduction assays were conducted on *M. truncatula* WT ecotype R108 and A17 and *dnf2–4* (R108 genetic background), and *dnf2-1*, *dnf1* mutants (A17 genetic background) inoculated with *S. medicae* strain WSM419. A Kruskal-Wallis one-way ANOVA test and a post-hoc Tukey’s test were performed and statistically identical values were attributed identical letters (n = 3). (b) Acetylene reduction assay was conducted on *M. truncatula* WT ecotype A17 and *dnf2-1* mutant inoculated with strain WSM419 alone (control) or in combination with *E. coli* K12, *Bradyrhizobium* sp. ORS285 or *P. fluorescens* Q2–87. Plants were grown under *in vitro* conditions on BNM supplemented with either agar or agarose and analyzed at 14 dpi (a) or with Agar and Phytagel and analyzed at 25 dpi (b). A Mann-Whitney test was performed between WT and *dnf2-1* mutant for each condition. The star indicates a significant difference (p-value <0.05) (n = 4) Error bars represent standard errors.

In order to determine if *dnf2–4* plants producing pink pigmented nodules correspond to symbiotically efficient nodules, we performed acetylene reduction assay (ARA) on WT and *dnf2–4* plants grown on agar- and on agarose-based media. The assays were performed using the *S. medicae* strain WSM419 which, in contrast to the RM41, is able to fix nitrogen in nodules of both A17 and R108 ecotypes of *M. truncatula*. The results showed that the agarose-based medium restored the capacity to reduce acetylene in the *dnf2–4* mutant (Figure 3a). As the *dnf2–4* allele is in the ecotype R108 background, we also evaluated the acetylene reduction activity in the *M. truncatula* A17 background with the *dnf2-1* mutant derived from A17. This *dnf2-1* line also recovered its nitrogen fixation capacity when grown on agarose based-medium (Figure 3a) demonstrating that the conditional phenotype of *dnf2* is not restricted to *dnf2* mutants of the R108 ecotype.

In order to determine if the conditional phenotype is a general feature of fix^−^ mutants, the previously described A17 *dnf1* fix^−^ line [21], [23] was tested for its symbiotic capacity on agarose-based medium. *dnf1* remained unable to reduce acetylene on agarose-based medium (Figure 3a) indicating that the observed conditional phenotype of *dnf2* is not a general feature of fix^−^ mutants. Additional analyses revealed that nitrogen fixation is also restored in *dnf2* when plants are cultivated on medium solidified with Phytagel, another agar-substitute, extracted from bacteria (Figure 3b). It should be noted that the rescue of *dnf2* nitrogen fixation varies from one experiment to the other and the presented data indicate that in some conditions, *dnf2* symbiotic capacity are similar to the WT. Together, these results indicate that plant growth conditions, represented here by the different types of solidifying agents, can overcome the requirement of *DNF2* for nitrogen fixation in R108 and A17 ecotypes.

### *dnf2* Functional Nodules Correctly Express Symbiotic Markers and do not Express Defense Genes

In order to determine if nitrogen fixation rescue is associated with the loss of defense-like reactions and with the recovery of normal nodulin expression in the *dnf2* functional nodules, RT-qPCR analysis were performed. Expression of defense markers, *PR10*, *PAL*, *chitinase*, *NDR1*, *VSP*, and of *Leghemoglobin* and *NCR001*, *NCR006*, *NCR094*, *NCR096*, *NCR109* and *NCR121* were evaluated in *dnf2* necrotic nodules developed on agar-based media and in *dnf2* functional nodules developed on Phytagel-based media as well as in WT nodules grown in the same conditions. Defense markers were not induced as compared to the WT in *dnf2* nodules developed under permissive condition (Figure S1a) and expression of the symbiotic markers was normal (Figure S1b,c). In contrast, in *dnf2* nodules developed under restrictive conditions, defense markers were induced as compared to the WT whereas *Leghemoglobin*, as well as of all tested *NCR* genes expressions were drastically reduced (Figure S1) when compared to WT nodules. Together, these data suggest that WT phenotype is completely recovered in functional *dnf2* pinkish nodules developed under permissive condition.

### Plant Growth Effect on the *dnf2–4* Phenotype is Transient

**Figure 4:**
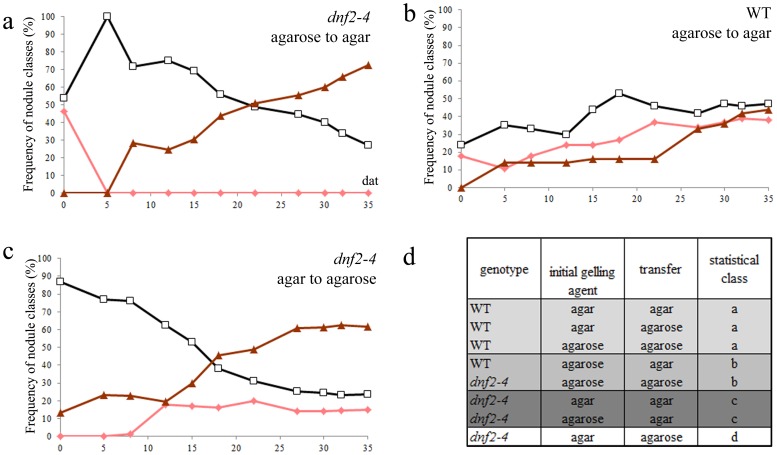
Influence of the plant growth conditions on *dnf2* phenotype is transient. (a–c) Frequencies of nodule classes after transfer to agar or agarose medium. *M. truncatula dnf2–4* and WT plants (n = 24 for every conditions) inoculated with *S. meliloti* Rm41 were cultivated *in vitro* on BNM using agar or agarose as a gelling agent for 14 days and transfer to new medium with the same or different gelling agent. Pink nodules are represented by diamonds, white nodules by open squares and brownish nodules by triangles. The experiment was repeated three times with similar results. (d) analysis of the distribution of nodule classes at 35 days after transfer. Statistically identical distribution are attributed identical letters (Chi-Square Test of Homogeneity with Bonferroni correction, p-value = 2.2–16).

In order to determine if plant substrates provoke the irreversible inability to fix nitrogen in *dnf2*, *S. medicae* strain WSM419-inoculated WT and *dnf2* plants were cultivated on agar- and agarose-based media and transferred once the first mature nodules were observed (14 dpi) on agar- or agarose-based medium. The frequencies of the different classes of nodules (white, pink, brown) were then monitored (Figure 4, Figure S2). The transfer from agarose to agar medium results in an increase in the formation of necrotic nodules irrespective of the genetic background (Figure 4a and b). WT plants transferred from agarose to agar based medium also triggered the formation of brownish nodules (Figure 4b). This suggests that the growth on agar medium is more stressful and that plants grown on agar need to adapt to this condition. This is not observed when WT plants were transferred to identical medium, indicating that plants are already adapted (Figure 4d). No pink nodules developed after the transfer of *dnf2–4* plants to agar-based medium, regardless of the initial gelling agent (Figure 4a, Figure S2). The pink nodules initially formed by the *dnf2–4* mutant on agarose based medium turned to white or brownish within the first five days after transferred to agar-based medium (Figure 4a). In contrast, the appearance of pink nodules only occurred when *dnf2–4* plants were transferred onto agarose-based medium regardless of the initial growth medium (Figure 4c, Figure S2). These results show that the plant substrate effect on *dnf2* is reversible and can trigger the conversion of fixing to non-fixing plants and vice-a-versa.

### Plant Substrate Effect on the *dnf2–4* Phenotype Acts at Distance

**Figure 5:**
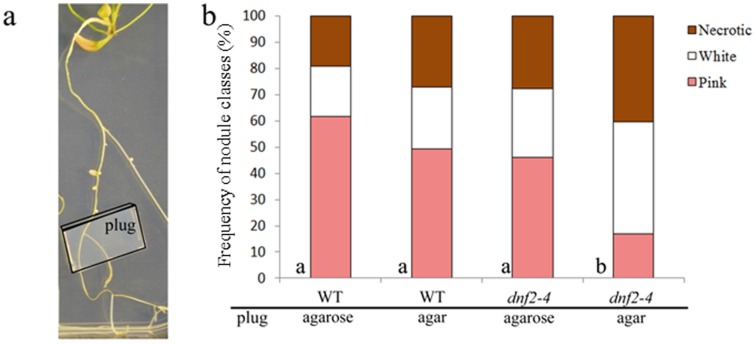
The plant substrate triggers *dnf2* fix^−^ phenotype at distance. *dnf2–4* and WT plants nodulated by *S. meliloti* Rm41 were grown on agarose-based BNM. Agar- or agarose-based plugs (1.5×1×0.5 cm) were laid onto root systems at 15 dpi and the color and numbers of nodules produced by 24 plants were monitored. The experimental set up is illustrated in (a). (b) Distribution of nodule classes 35 days after plug addition for WT and *dnf2–4* grown on agarose based medium. The experiment was repeated three times with similar results. Statistically identical distribution are attributed identical letters (Chi-Square Test of Homogeneity with Bonferroni correction, p-value = 1.32e-06).

To determine if the agar-based medium can trigger the fix^−^ phenotype at distance, WT and *dnf2–4* plants were cultivated on both agar- or agarose-based BNM medium. 15 days post inoculation, agar- or agarose-based BNM medium plugs were placed onto root systems of the wild-type and *dnf2–4* plants at a distance from the nodules (Figure 5a). The number and the color of the nodules were then monitored (Figure S3). At 35 days after placing the plugs, an agarose-based BNM plug did not stop the development of pink nodules in the *dnf2–4* mutant grown on agarose-based medium (Figure 5b). In contrast, an agar-based BNM plug significantly modified the distribution with a decrease in the number of pink nodules and an increase of the brownish nodules for *dnf2–4* plants grown on agarose-based medium (Figure 5b). 35 days after placing the agarose or agar plugs, the proportion of white, brown and pink nodules were similar in the WT plants grown on agarose. These results indicated that the plant growth condition effect on the *dnf2–4* fix^−^ phenotype results from a signal that can act distantly.

### The *dnf2* Fix^−^ Phenotype is not Restored by Addition of Standard Defense Priming Agents

Based on the above results we hypothesize that component(s) present in the agar but absent in the agarose might trigger or prime defense reactions in the *dnf2–4* nodules. In order to determine if the addition of molecules known to trigger defense reactions can restore the *dnf2* fix^−^ phenotype on permissive conditions we supplemented the medium with plant defense elicitors or phytohormones involved in defense reactions. The pattern-triggered immunity (PTI) activator, Flg22, and the systemic acquired resistance messengers, salicylic and jasmonic acids (SA and JA, respectively) did not restore the fix^−^ phenotype of *dnf2–4* on agarose-based medium (Table S2). In addition to these two molecules, we aimed at creating a more complex environment potentially priming or triggering defenses in *dnf2* nodules. To do this, the presence of yeast extract or simple artificial microflora during the symbiotic interaction was also evaluated. To test the effect of simple microflora, co-inoculations of *S. medicae* WSM419 with the enteric bacteria *E. coli* K12, the rhizobium *Bradyrhizobium sp.* ORS285 [32] and the induced systemic resistance (ISR) inducing bacterium *Pseudomonas fluorescens* Q2–87 [30] were performed. None of these strains restored the *dnf2* fix^−^ phenotype as determined by nodule color or ARA measurements at 25 dpi (Figure 3b, Table S2) in the agarose grown *dnf2–4* plants. Similar results were observed when the medium was supplemented with yeast extract that contain the PTI activator chitin (Table S2).

### The *dnf2* Fix^−^ Phenotype is Triggered by the Complex Defense Priming Agent Ulvan

**Figure 6:**
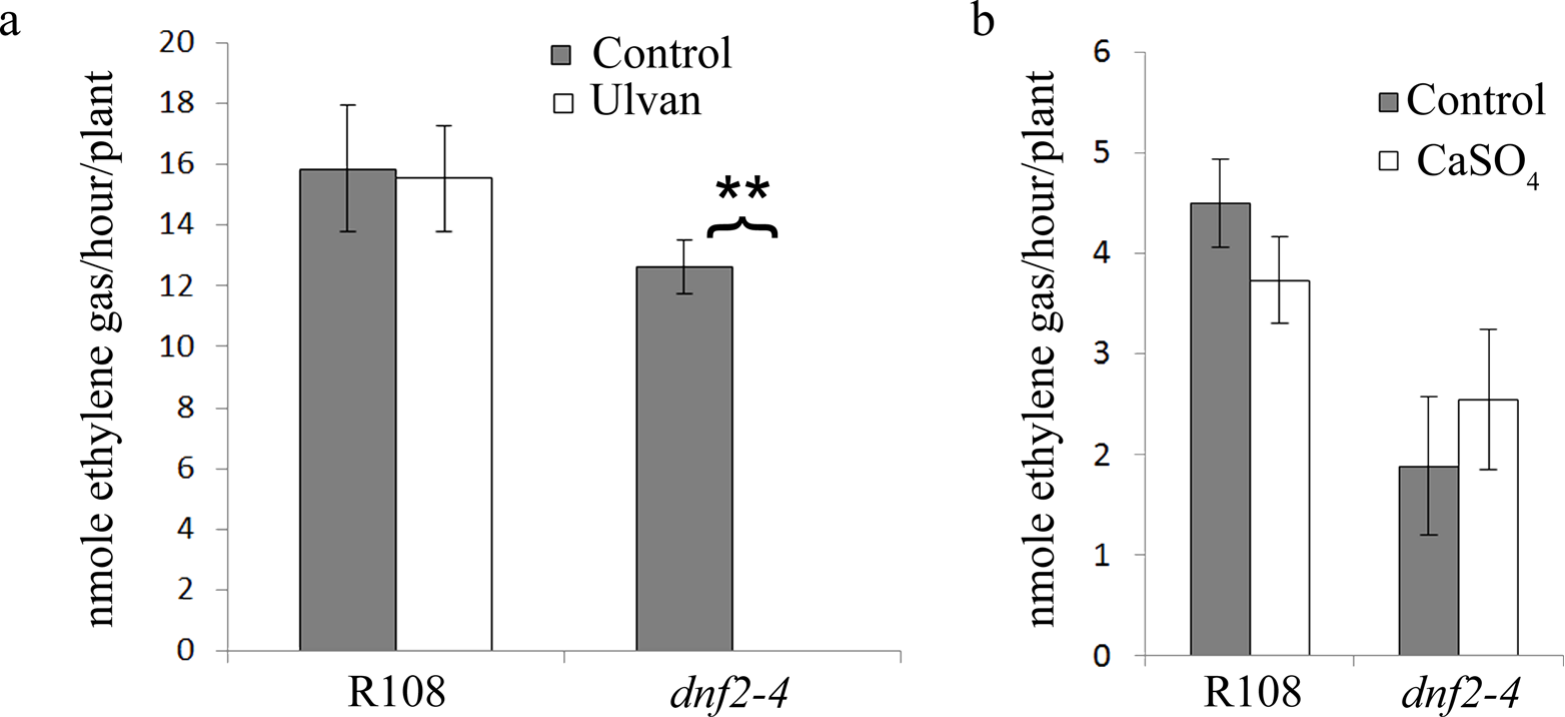
Ulvan abolishes *dnf2* nitrogen fixation on permissive condition. *M. truncatula* WT R108 and *dnf2–4* plants were cultivated on agarose BNM supplemented or not with 1% ulvan. Acetylene reduction assays were conducted on plants 27 dpi with *S. medicae* WSM419 (n = 8) (a). *M. truncatula* WT R108 and *dnf2–4* plants were cultivated on Phytagel BNM supplemented or not with 10 mM CaSO_4_. Acetylene reduction assays were conducted on plants 14 dpi with *S. medicae* WSM419 (n = 5) (b). A Mann-Whitney test was performed between WT and *dnf2-1* mutant for each condition. Stars indicate significant differences (** p-value <1e-03) Error bars represent standard errors.

Ulvan is a complex polysaccharide extracted from algae known to prime plant defenses in *Arabidopsis* and *M. truncatula* [43], [44]. We tested its capacity to restore the fix^−^ phenotype of *dnf2* on agarose grown plants. Addition of 1% ulvan in the agarose-based medium did not trigger any detectable change in the symbiotic capacity of the WT plants based on nodule histology and acetylene reduction capacity (Figure 2c,f,i; Figure 6a; Figure S4). In contrast, 1% ulvan restored the *dnf2* phenotype (fix^−^ nodules) in the *dnf2–4* mutant grown on agarose-based medium (Figure 2l,o,r; Table S2). Further, using ARA assay, we showed that addition of ulvan in the agarose-based medium triggered *dnf2* fix^−^ phenotype in *dnf2–4* mutant grown on permissive condition (Figure 6a).

In order to determine if *DNF2* expression is regulated by the plant growth conditions, we analyzed *DNF2* expression in the nodules developed on R108 plants cultivated on permissive agarose- and Phytagel-based BNM or restrictive agar-based BNM and agarose-based BNM supplemented with ulvan using RT-qPCR analysis. No significant differences were observed in the abundance of *DNF2* transcript between the tested conditions (Figure S5a). To determine if *DNF2* expression pattern is modified by the permissive/restrictive conditions, R108 transgenic plants expressing the β-glucuronidase under the promoter of *DNF2* were used [5]. Nodules from plants cultivated on agarose- and agar–based medium were analyzed. *DNF2* expression was essentially detected in the infection zone in both conditions (Figure S5b,c).

In contrary to agarose and Phytagel, a high content of sulfate is a striking feature common to agar and ulvan. This difference in the sulfate content [45] or the presence of sulfate ions in the medium could be responsible for a different response of the mutant to the different compounds. To test this possibility, a Phytagel based medium was supplemented with 10 mM CaSO_4_ (a concentration similar to that found in ulvan [46]). However, the *dnf2* mutant did not recover its fix^−^ phenotype in presence of 10 mM CaSO_4_ (Figure 6b), indicating that high sulfate concentration alone is not enough to trigger the *dnf2* fix^−^ phenotype. Altogether these results suggest that *DNF2* expression is not significantly modified by plant growth conditions and that the fix^−^ phenotype of the *dnf2* mutant is triggered by ulvan treatment through defense priming.

## Discussion

*M. truncatula dnf2* mutant lines were isolated during independent genetic screens as fix^−^ mutants [5], [21], [22], [23], [47]. In the *dnf2* alleles, the nodule organogenesis is not altered and the symbiotic process is blocked only after bacterial release from the infection thread [5]. Defense-like reactions in the nodules and defect in bacteroid differentiation were reported in these mutants [5], [6]. It remained unclear whether differentiation defect is a cause or a consequence of defense-like reactions. Here we report that the *bacA* mutant does not trigger defense-like reactions (Figure 1). Furthermore, we show that *dnf2* KO mutants can form a functional symbiosis when grown *in vitro* on agarose- and Phytagel-based media (Figure 3a,b). We show that this trait is not a general feature of the fix^−^ mutants as the *dnf1* line remains unable to reduce nitrogen under *dnf2* permissive conditions (Figure 3a).

Our study further shows that addition of ulvan converts the condition from permissive to restrictive for the *dnf2–4* symbiotic phenotype on agarose and Phytagel-based media. Ulvan is a complex carbohydrate polymer extracted from the green algae *Ulva spp.* [46]. In *M. truncatula*, it induces the expression of a *PR10* gene as well as a broad range of defense-related genes, notably genes involved in phytoalexin biosynthesis and cell wall proteins [43]. Interestingly, we found that the *PR10* gene was expressed at high level in *M. truncatula* roots grown on agar-based BNM medium and this expression was strongly reduced in wild-type nodules while in nodules of the *dnf2–4* mutant, the *PR10* gene was significantly expressed [5]. Ulvan activates defense priming through the JA pathway on both *M. truncatula* and *Arabidopsis thaliana* [44] and induces resistance against the pathogenic fungus *Colletotrichum trifolii* [43]. In wheat and barley ulvan also acts as a priming agent allowing resistance against *Blumeria graminis* [48]. The ulvan effect observed here on *dnf2* phenotype thus confirms the role of *DNF2* in symbiotic repression of plant defenses at the stage of the bacterial internalization.

Our study also shows that agar triggers transiently and at distance the inability of the *dnf2* mutants to reduce nitrogen (Figure 4,5). The compound(s) in agar, responsible for the *dnf2* fix^−^ phenotype, can act at a distance and only transiently (Figure 4,5). This suggests that *in planta* the signal triggering the fix^−^ phenotype (including defense-like reactions) is mobile or alternatively the agar mobile element could be a small molecule released into the medium or the plant atmosphere. It also suggests that *DNF2* prevents the fix^−^ phenotype triggered by a mobile signal. This observation is reminiscent of defense priming.

The way how plants perceive microbial invaders and the signaling cascades controlling the activation of defense reactions are now relatively well characterized in *Arabidopsis* [49], [50]. It is known that microbial associated molecular patterns (MAMPs) such as Flg22, the flagellin active epitope, are able to trigger defense reactions on a wide variety of plants [51]. Hormone signaling enhancing resistance to a variety of pathogens has also been described and JA and SA act in these processes [18] referred to as induced systemic resistance (ISR) and systemic acquired resistance (SAR), respectively. However, we found that neither Flg22, SA, JA nor ISR were able to switch the condition from permissive to restrictive for *dnf2*. Considering the ulvan effect and its presumed mode of action through the JA pathway, it is unexpected that JA addition does not restore the *dnf2* fix^−^ phenotype under permissive conditions. This could reflect difference(s) in the regulatory network in *M. truncatula* in which, in contrast to *Arabidopsis*, knowledge on signaling cascades leading to plant defenses remains poorly understood. Genetic tools will help elucidate these potential differences.

Determining the biochemical function of DNF2 will be a key step in the comprehension of the mechanisms allowing plant tolerance to rhizobia. DNF2 displays similarity with phosphatidyl-inositol phospholipase C (PI-PLC) X-domain [5]. In addition to the X-domain, experimentally described plant PI-PLCs harbor a calcium binding domain and a so called Y-domain containing residues of the catalytic site [52]. The DNF2 atypical structure makes difficult to predict its biochemical function despite that bacterial PI-PLCs containing only the X-domain were shown to be functional and to be able to cleave phosphatidyl-inositol and/or GPI anchors [53]. A recent study suggests that human PI-PLCXD containing protein also displays phospholipase activity while laking the Y and Ca^2+^ binding domains [54], leaving open the possibility that PI-PLCXD containing proteins, amongst which is DNF2, play a role in phospholipid cleavage. Without any demonstrated biochemical activity, for now, it is only possible to speculate why DNF2 is unnecessary under permissive condition. It seems resonnable to speculate that the DNF2 substrate or ligand is absent in the cells under permissive conditions and that this substrate or free ligand is responsible for the plant defense activation. Irrespective of the DNF2 biochemical function, homologues of this protein are present in all plant species (including *Arabidopsis* and major crop species) [5] suggesting a more general role in plants than its action during symbiosis. The potential involvement of these proteins in the tolerance to endophytic microbia remains to be investigated. In this respect, the identification of several indigenous rhizobia in *Arabidopsis* root microbiota should make it possible to investigate this proposed function [55], [56].

From the work presented here, using artificial *in vitro* growth conditions, we evidenced a mechanism that allows the development of a functional nodule in the *dnf2* mutant. We propose a hypothetical model for the action of DNF2 in the control of bacteroids persistence in the symbiotic organ. In this hypothetical model presented in Figure S6, we propose that the *DNF2* restrictive conditions prime defense reactions in the plant but this elicitation is counteracted by the action of DNF2. This would explain the reason why when plants are cultivated under these conditions, the rhizobial infection triggers defense-like reactions in the *dnf2* mutant but not in the WT. In this hypothetical model, under priming conditions, in absence of DNF2 the rhizobial infection results in defense elicitations and death of the rhizobia. In growth conditions which do not prime the defense reactions (*i.e.* permissive conditions), the effect of the rhizobial infection alone is not able to reach the threshold that results in defense reactions and the symbiotic interaction can take place. Determining the DNF2 biological function will suggest validity of this model.

## Supporting Information

Figure S1 ***dnf2–4*** **pink nodules correctly express symbiotic markers and do not express defense genes.** Expression of defense (panel a) marker and of symbiotic markers (panels b and c) were evaluated by qRT-PCR in *dnf2–4* nodules (21 dpi) induced by *S. medicae* strain WSM419. Data were normalized with *MtACTIN* expression and reported to the expression in WT nodules developed in the same conditions.
(TIF)

Figure S2 **Plant growth conditions effect on** ***dnf2*** **plants is reversible.** (a–e) Frequencies of nodule classes after transfer to agar or agarose media. *M. truncatula dnf2–4* and WT plants (n = 24 for every conditions) inoculated with *S. meliloti* Rm41 were cultivated *in vitro* on BNM using either agar or agarose as a gelling agent for 14 days and transfer to new medium with the same or a different gelling agent. Pink nodules are represented by diamonds, white nodules by open squares and brownish nodules by triangles. The experiment has been repeated three times with similar results. (f) % of nodule classes at 35 days after transfer.
(TIF)

Figure S3 **Plant substrates effect on** ***dnf2*** **can act at distance.** *M. truncatula* R108 and *dnf2–4* plants (n = 24 plants for every condition) nodulated with *S. meliloti* Rm41 were grown on agarose based BNM. Agar- or agarose-based BNM plugs (1.5×1×0.5 cm) were laid onto root systems of the plants 15 dpi. The y-axis represents the % of nodule classes. Abscises represent days after addition of the plug. The experiment has been repeated three times with similar results.
(TIF)

Figure S4 **Gelling agents do not alter WT nitrogen fixation capacity.** Acetylene reduction assays were conducted on *M. truncatula* WT R108 plants cultivated on BNM solidified with the indicated gelling agents, 21 dpi with *S. medicae* WSM419. A Kruskal-Wallis one-way ANOVA test did not show significant differences between conditions (p-value = 0.3349).
(TIF)

Figure S5 ***DNF2*** **expression is not controlled by DNF2 requirement conditions.** *DNF2* expression level and expression pattern were investigated in nodules of *M. truncatula* WT R108 (A) plants or transgenic WT pDNF2::Gus (B,C). The plants were cultivated on BNM solidified with the indicated agent and *DNF2* expression level was followed by RT-qPCR using *Actin* as a reference (A). Results are expressed as ratio versus expression on Phytagel based BNM. Error bars represent the standard error on three biological repetitions. Transgenic plants expressing the reporter construct were cultivated on Agar- (B) and agarose-BNM (C), scales bars represent 100 µm.
(TIF)

Figure S6 **Hypothetical model for the effect of growth conditions on DNF2 requirement for symbiosis.** In *M. truncatula* WT and *dnf2* nodules from plants cultivated on non-defense priming environments (agarose- and Phytagel-based media), the defense elicitation does not reach the threshold for defense reactions and the symbiosis is efficient (central part of the figure). When plants are cultivated on defense priming environments (agar- and ulvan supplemented agarose-media) elicitation reaches the threshold for defenses (left and right part of the figure) but, in the WT nodules, DNF2 (represented by a green box) prevents defense reactions to a large extent. In contrast, the *dnf2* mutant nodules develop defense reactions.
(TIF)

Table S1 **List of primers used during this study.**
(TIF)

Table S2 **Ulvan triggers the** ***DNF2*** **requirement for symbiosis.**
(TIF)
