# Supplementary figures and images for: Growth Conditions Determine the DNF2 Requirement for Symbiosis
Source: PLoS One. 2014 Mar 14;9(3):e91866. doi: 10.1371/journal.pone.0091866 (PMC3954807; doi:10.1371/journal.pone.0091866)

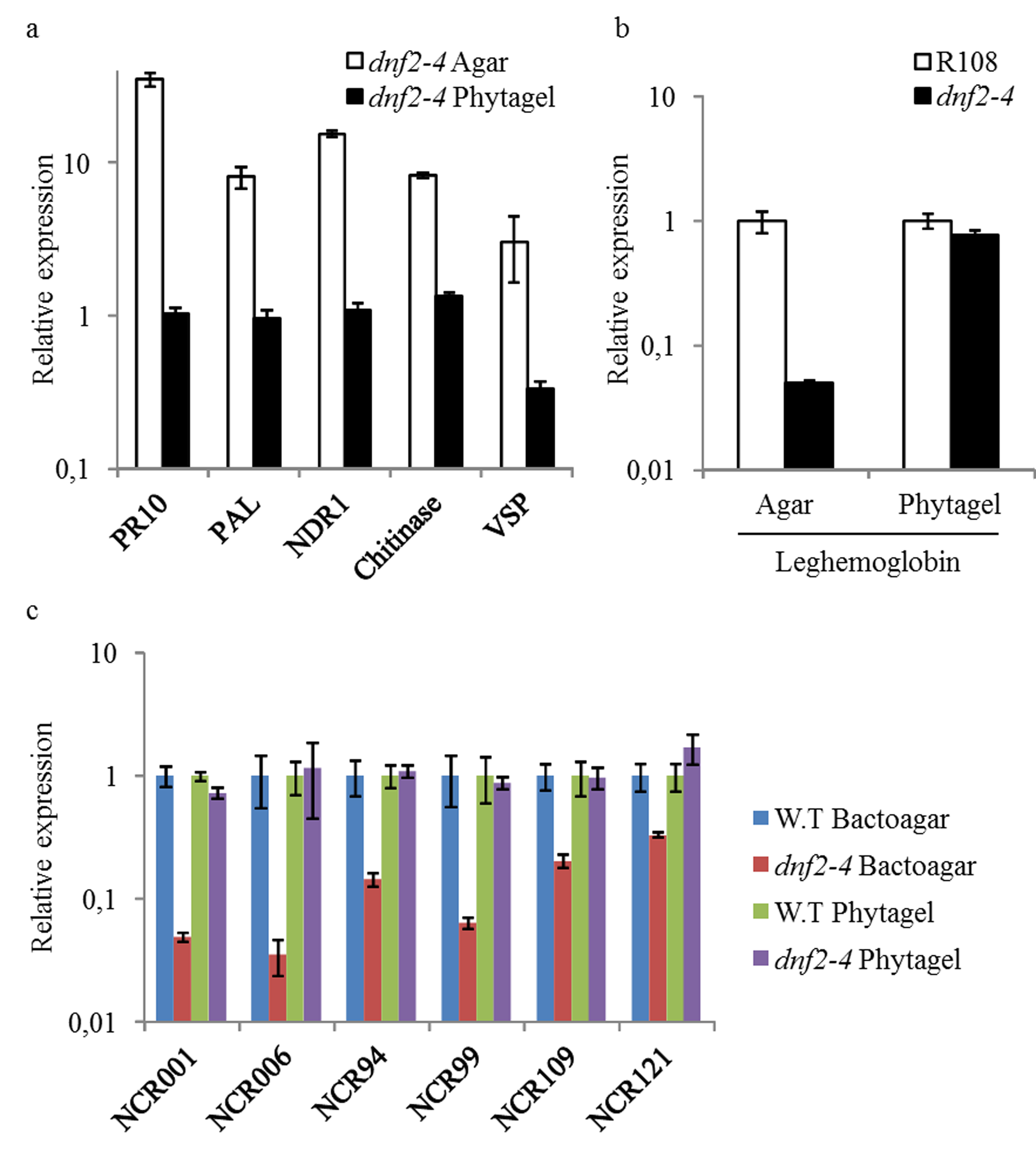

Supplement: Figure S1 — dnf2–4 pink nodules correctly express symbiotic markers and do not express defense genes. Expression of defense (panel a) marker and of symbiotic markers (panels b and c) were evaluated by qRT-PCR in dnf2–4 nodules (21 dpi) induced by S. medicae strain WSM419. Data were normalized with MtACTIN expression and reported to the expression in WT nodules developed in the same conditions. (TIF) [file pone.0091866.s001.tif]

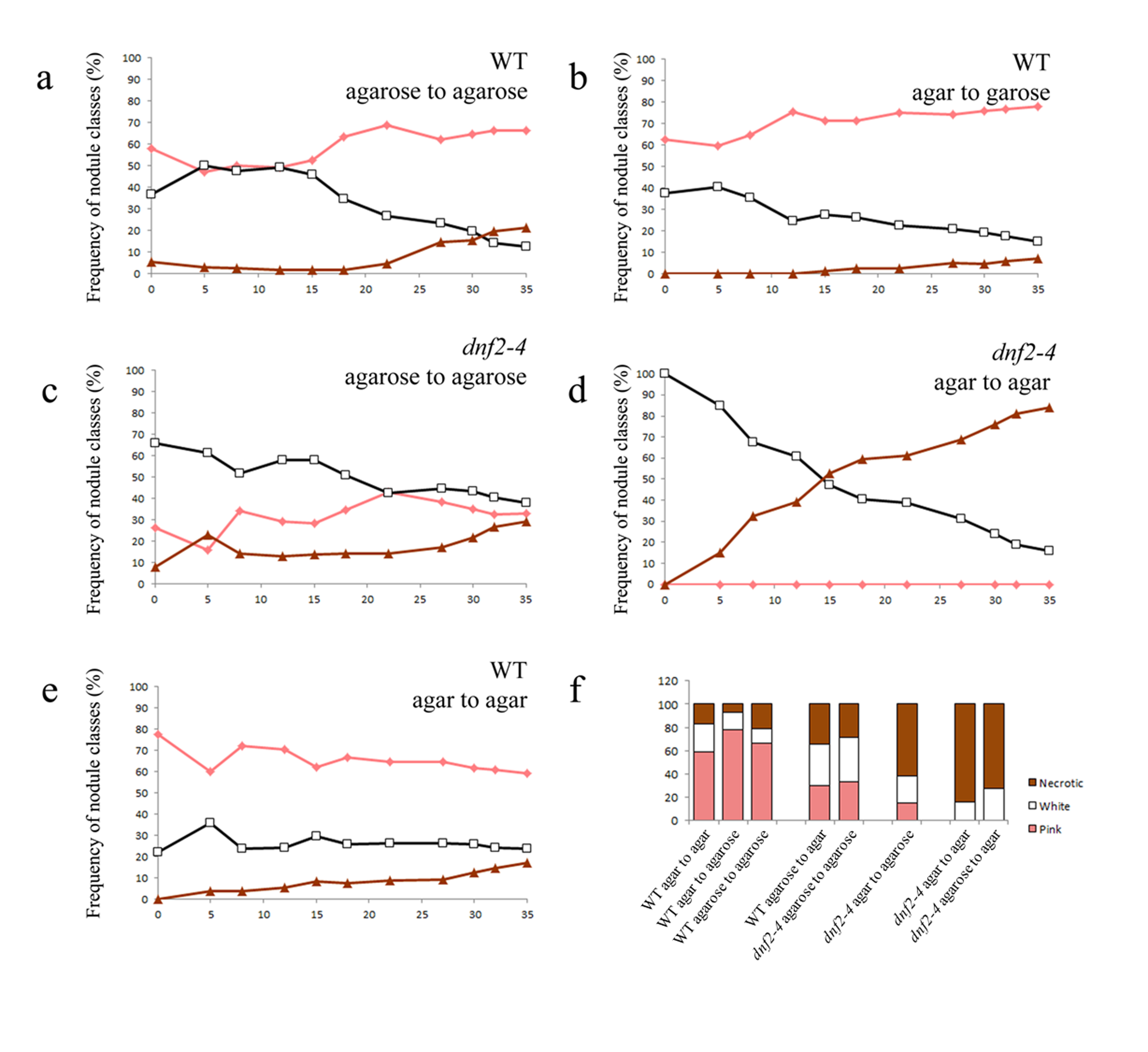

Supplement: Figure S2 — Plant growth conditions effect on dnf2 plants is reversible. (a–e) Frequencies of nodule classes after transfer to agar or agarose media. M. truncatula dnf2–4 and WT plants (n = 24 for every conditions) inoculated with S. meliloti Rm41 were cultivated in vitro on BNM using either agar or agarose as a gelling agent for 14 days and transfer to new medium with the same or a different gelling agent. Pink nodules are represented by diamonds, white nodules by open squares and brownish nodules by triangles. The experiment has been repeated three times with similar results. (f) % of nodule classes at 35 days after transfer. (TIF) [file pone.0091866.s002.tif]

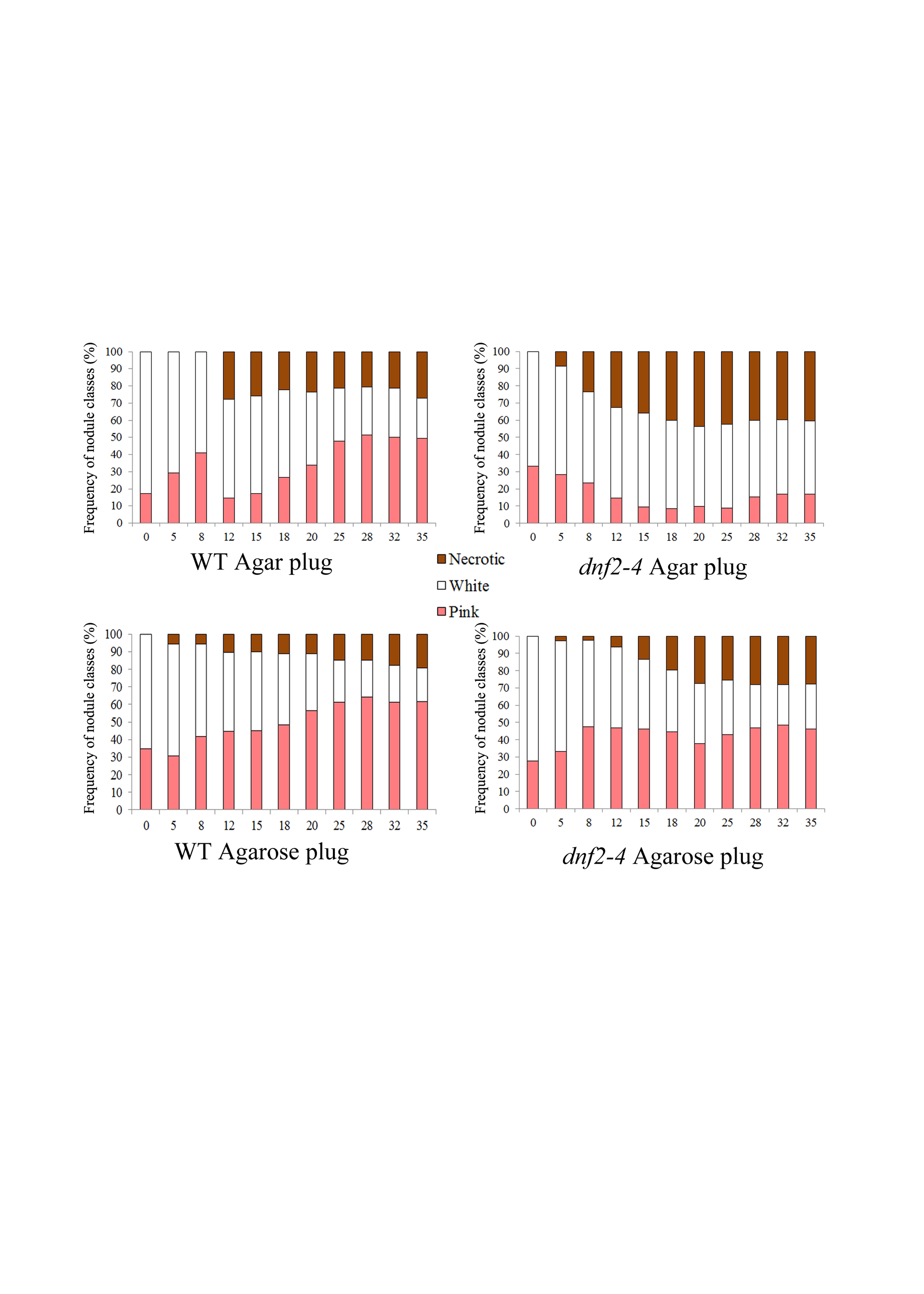

Supplement: Figure S3 — Plant substrates effect on dnf2 can act at distance. M. truncatula R108 and dnf2–4 plants (n = 24 plants for every condition) nodulated with S. meliloti Rm41 were grown on agarose based BNM. Agar- or agarose-based BNM plugs (1.5×1×0.5 cm) were laid onto root systems of the plants 15 dpi. The y-axis represents the % of nodule classes. Abscises represent days after addition of the plug. The experiment has been repeated three times with similar results. (TIF) [file pone.0091866.s003.tif]

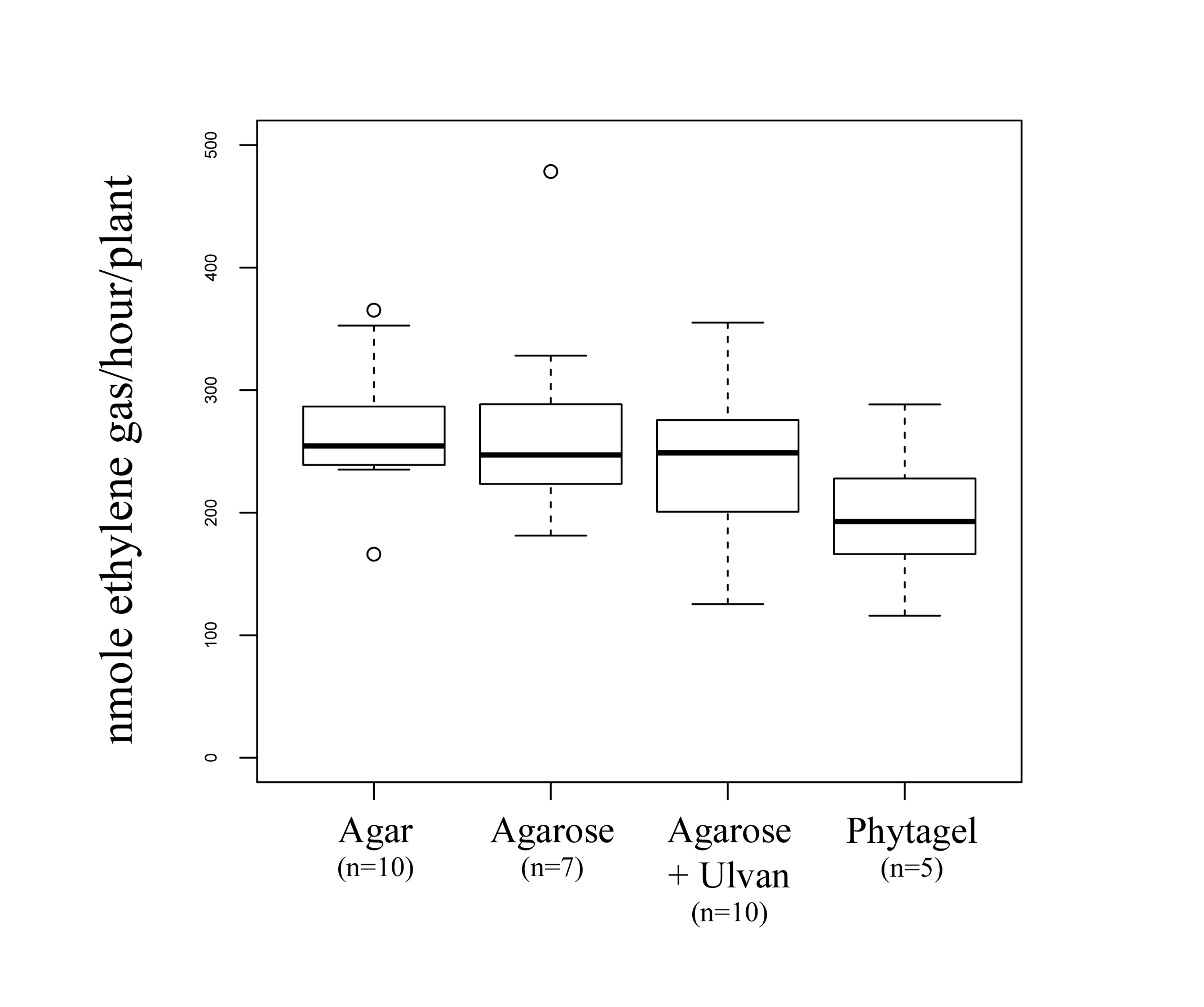

Supplement: Figure S4 — Gelling agents do not alter WT nitrogen fixation capacity. Acetylene reduction assays were conducted on M. truncatula WT R108 plants cultivated on BNM solidified with the indicated gelling agents, 21 dpi with S. medicae WSM419. A Kruskal-Wallis one-way ANOVA test did not show significant differences between conditions (p-value = 0.3349). (TIF) [file pone.0091866.s004.tif]

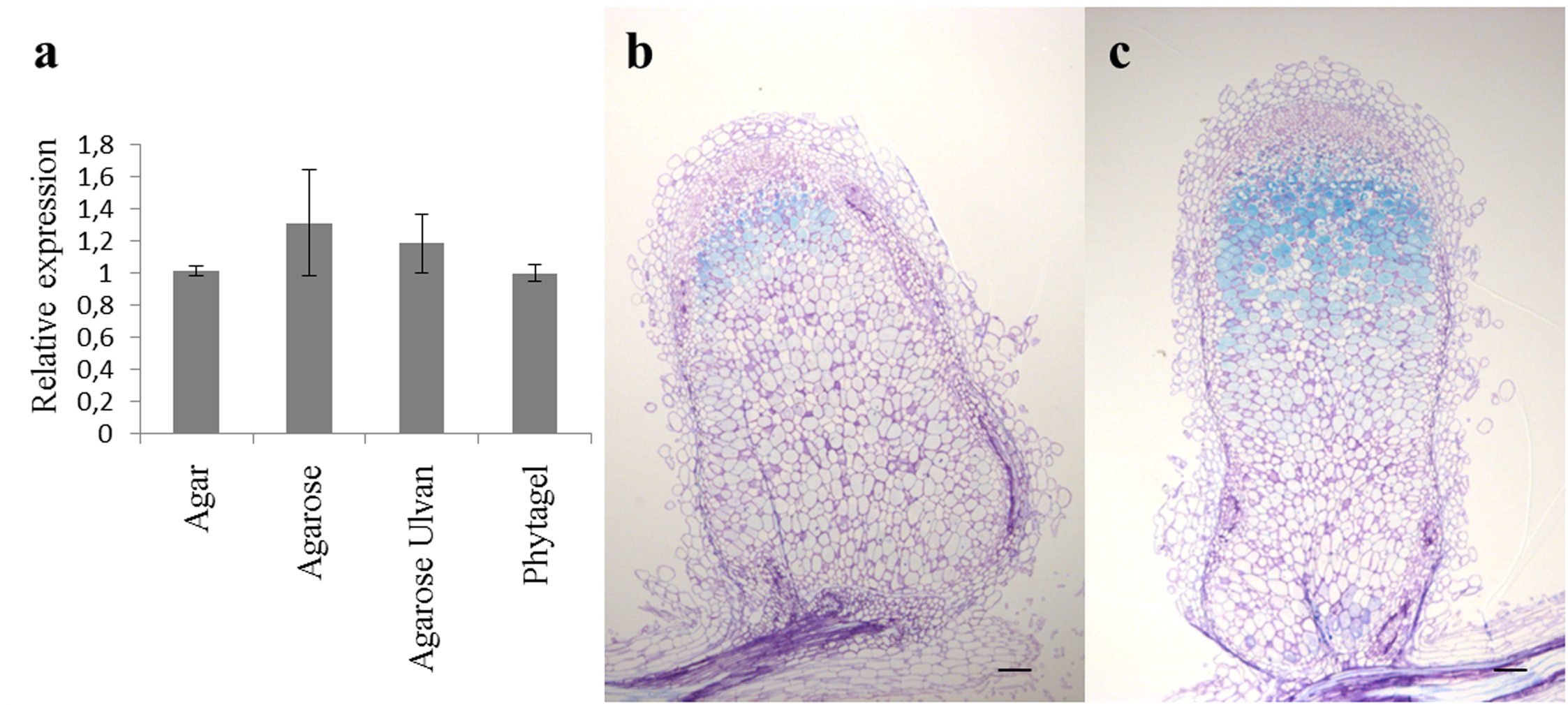

Supplement: Figure S5 — DNF2 expression is not controlled by DNF2 requirement conditions. DNF2 expression level and expression pattern were investigated in nodules of M. truncatula WT R108 (A) plants or transgenic WT pDNF2::Gus (B,C). The plants were cultivated on BNM solidified with the indicated agent and DNF2 expression level was followed by RT-qPCR using Actin as a reference (A). Results are expressed as ratio versus expression on Phytagel based BNM. Error bars represent the standard error on three biological repetitions. Transgenic plants expressing the reporter construct were cultivated on Agar- (B) and agarose-BNM (C), scales bars represent 100 µm. (TIF) [file pone.0091866.s005.tif]

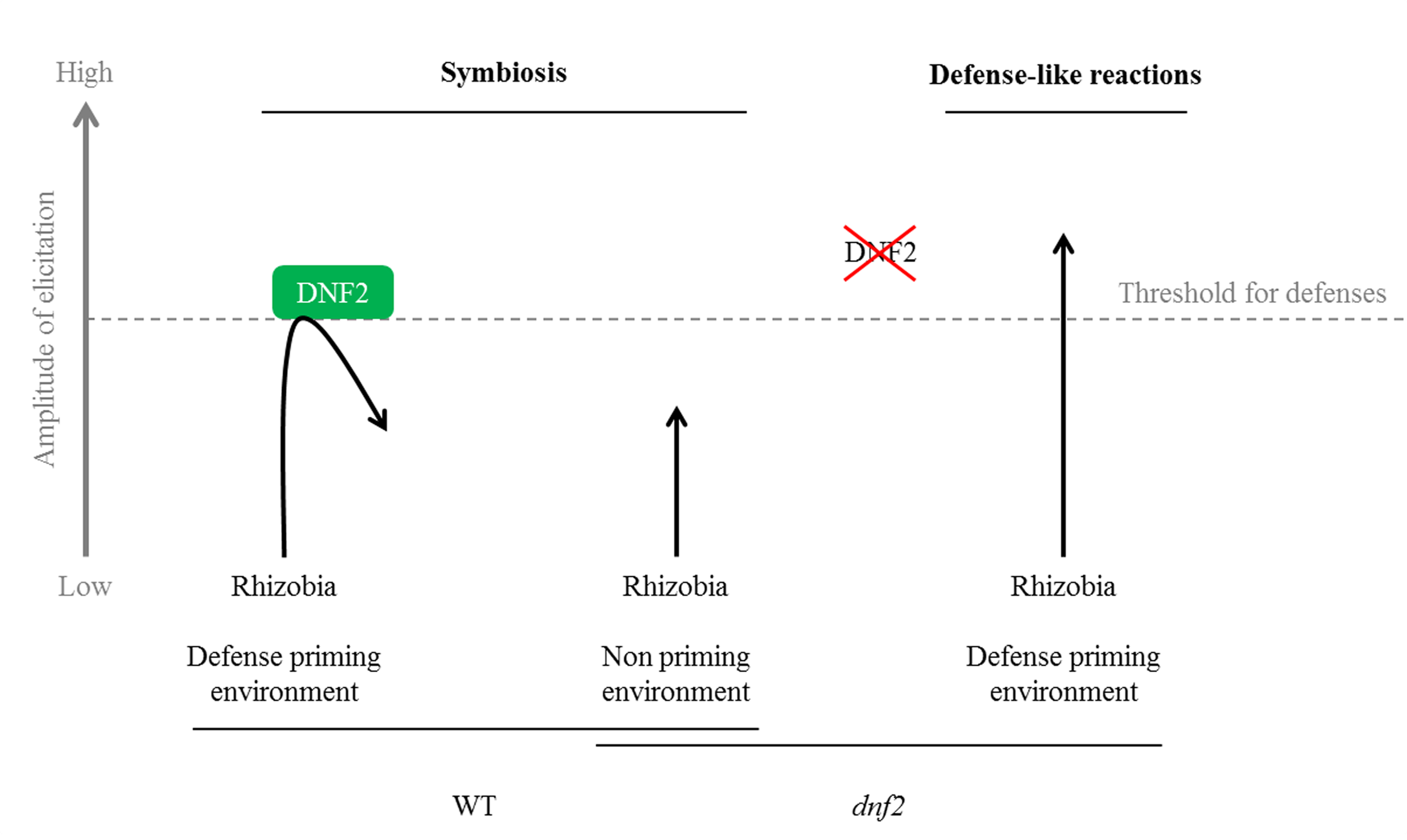

Supplement: Figure S6 — Hypothetical model for the effect of growth conditions on DNF2 requirement for symbiosis. In M. truncatula WT and dnf2 nodules from plants cultivated on non-defense priming environments (agarose- and Phytagel-based media), the defense elicitation does not reach the threshold for defense reactions and the symbiosis is efficient (central part of the figure). When plants are cultivated on defense priming environments (agar- and ulvan supplemented agarose-media) elicitation reaches the threshold for defenses (left and right part of the figure) but, in the WT nodules, DNF2 (represented by a green box) prevents defense reactions to a large extent. In contrast, the dnf2 mutant nodules develop defense reactions. (TIF) [file pone.0091866.s006.tif]

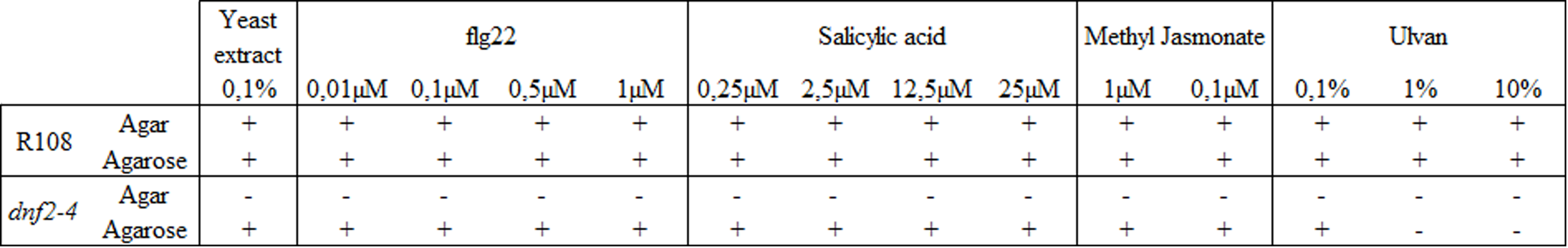

Supplement: Table S2 — Ulvan triggers the DNF2 requirement for symbiosis. (TIF) [file pone.0091866.s008.tif]
